# Ancient DNA analyses of remains of the Medici family (16^th^ century) provide insights into the genetic variation of *Plasmodium falciparum*

**DOI:** 10.1016/j.isci.2026.116371

**Published:** 2026-06-17

**Authors:** Alexander Ochoa, Samantha L. Miller, Patrick F. Reilly, Gino Fornaciari, Antonio Fornaciari, Giulia Riccomi, Valentina Giuffra, Adalgisa Caccone, Serena Tucci

**Affiliations:** 1 Department of Anthropology, Yale University, New Haven, CT 06511, USA; 2 Department of Ecology and Evolutionary Biology, Yale University, New Haven, CT 06520, USA; 3 Division of Paleopathology, Department of Translational Research and New Technologies in Medicine and Surgery, University of Pisa, 56126 Pisa, Italy

**Keywords:** Microbial genomics, Omics, Genomics, Archeology, History

## Abstract

We evaluated the presence of six *Plasmodium* species in osteological remains from Grand Duke Francesco I de' Medici (GDFIDM; 1541–1587) and Cardinal Giovanni de' Medici (CGDM; 1543–1562). Targeted enrichment sequencing for mitochondrial genomes recovered 1,865 and 185 base pairs (bp) of *P. falciparum* from CGDM and GDFIDM, respectively, and 43 bp of *P. malariae* from GDFIDM. The CGDM *P. falciparum* sequence represents a previously uncharacterized haplotype with two unique mutations in intergenic and cytochrome *c* oxidase subunit 1 loci. Network analyses using nine additional ancient samples (Iron Age– 1940s) suggest that this haplotype is closely related to six sequences from Europe, Taiwan, and the Caribbean; the haplotype likely originated from a demographic expansion in Europe. Our study discovered a previously uncharacterized *P. falciparum* strain from the Italian Renaissance, increasing our knowledge of the diversity of this species and underscoring the use of ancient DNA for the detection and diagnosis of malaria.

## Highlights

• Detected ancient *Plasmodium* DNA in 16^th^ century Medici family osteological remains
• Grand Duke Francesco I de' Medici had traces of *P. falciparum* and *P. malariae*
• Cardinal Giovanni de' Medici had a previously uncharacterized *P. falciparum* strain
• The previously uncharacterized *P. falciparum* strain has two unique mutations

## Graphical abstract

**Figure:**
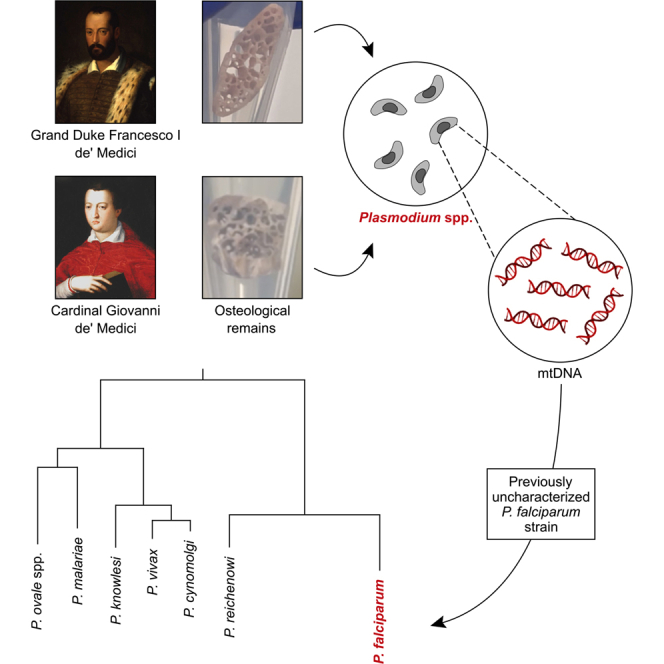

## Introduction

*Plasmodium falciparum*, the causal agent of the deadliest form of human malaria, is an obligate unicellular protozoan parasite transmitted by female anopheline mosquitoes. This protozoan conceivably proliferated in the agrarian communities of sub-Saharan Africa 5,000–4,000 years ago,^1^ where sizable human settlements and humid environmental conditions favored its transmission. Concomitant with human migrations, it remains unclear when *P. falciparum* spread to Europe. The earliest genetically confirmed case of *P. falciparum* in the continent dates to 371–176 Before Common Era (BCE) from Lower Austria.^2^ Furthermore, in Southern Italy, genetic evidence for the presence of *P. falciparum* dates to 1^st^–2^nd^ century Common Era (CE) skeletal remains.^3^^,^^4^

Malaria has played a defining role in the history of Italy, shaping its societies, economies, and landscapes from antiquity through the 20^th^ century.^5^^,^^6^^,^^7^^,^^8^ Ancient Greek and Roman authors, including Hippocrates and Celsus, provided some of the earliest descriptions of the disease, associating it with periodic fevers, weakness, and other symptoms recognizable today as malaria.^9^^,^^10^^,^^11^ In particular, the coastal plains, river valleys, and swampy areas of Tuscany were notorious for endemic malaria^12^; the disease was prevalent in low-lying and marshy areas like the Maremma, where stagnant water facilitated the breeding of mosquitoes, the vectors of the disease.^13^^,^^14^ During the Middle Ages and early Modern period, the disease remained endemic; major reclamation of marshes started only during the 18^th^–20^th^ centuries, culminating in the eradication campaigns of the 1950s when malaria was finally eliminated from Tuscany.^7^

Early work in paleomicrobiology at the turn of the 21^st^ century set the foundation for the detection of ancient pathogens from human remains, including dental pulp, bone, and mummified tissues.^15^^,^^16^^,^^17^ Advances in ancient DNA (aDNA) methods enabled the genetic analysis of ancient pathogens causing tuberculosis,^18^ syphilis,^19^ and vector-borne diseases like the plague^20^^,^^21^^,^^22^ and malaria.^2^^,^^3^^,^^4^^,^^23^^,^^24^ Recently, Michel et al.^2^ generated 13 ancient *P. falciparum* mitochondrial DNA (mtDNA) sequences from human remains in Europe, Asia, and the Americas, spanning ∼2,700 years of human history. These data complement two ancient *P. falciparum* mtDNA sequences, one from the 1^st^–2^nd^ century CE in Southern Italy,^3^^,^^4^ and another one from the 1940s in Northeastern Spain.^23^ However, to date, there is no information regarding the genetic variation of the *P. falciparum* strains circulating in Central Italy during the 16^th^ century. To fill this gap, we extracted aDNA from osteological remains belonging to two members of the Medici family, Grand Duke Francesco I de' Medici (hereafter “GDFIDM”; 1541–1587) and Cardinal Giovanni de' Medici (hereafter “CGDM”; 1543–1562), and used an in-solution DNA bait set targeting *Plasmodium* spp. mtDNA genomes. We focused on these Medici family members because their deaths were consistent with malarial infections, and their osteological remains were preserved within metal boxes at the Medici Chapels (Florence, Italy), ensuring favorable conditions for DNA extractions.

The two individuals analyzed, GDFIDM and CGDM, were siblings, born to the same father, Cosimo I de' Medici (first Grand Duke of Tuscany), and mother, Eleonora of Toledo. CGDM—along with his mother and another one of his siblings, Garzia—contracted malaria during a family trip to the coasts and marshes of Tuscany, well known at the time for being afflicted by “tertian fever.”^25^ Reportedly, these three members of the Medici family succumbed to severe fevers within a four-week span by the end of 1562.^25^ Twenty-five years later, GDFIDM (then Grand Duke of Tuscany) and his wife, Bianca Cappello, visited the Medici villa in Poggio a Caiano (Prato), which was located in a highly unsanitary rice-field area,^26^ a typical endemic malarial environment. The couple perished on consecutive days in 1587 after having manifested intermittent high fevers, consistent with malaria, prior to their deaths.^25^ Their sudden and near-simultaneous deaths, however, gave rise to rumors about poisoning with arsenic by GDFIDM’s brother, Cardinal Ferdinando; these claims were likely spread by courtiers familiar with the long-standing conflict between the brothers.^27^

Skeletal signs of malaria are not specific or diagnostic, as malaria primarily affects the soft tissues and blood; they include cranial porous lesions.^28^ Neither of these indicators were observed in the skeletal remains of GDFIDM and CGDM, presumably because the disease had a very rapid course. Previous immunological investigations indicate that GDFIDM and CGDM were both carriers of *P. falciparum*.^29^^,^^30^ However, to date, no genetic assessments have been made on their skeletal remains to confirm these findings.

In this study, we used aDNA methods to assess the presence of *Plasmodium* spp. in the osteological remains of the Medici family and to investigate levels of genetic variation in *P. falciparum*, examine phylogenetic relationships among ancient and modern strains of this species, and trace the spread and evolution of malaria in Central Italy during the Renaissance. This work contributes to expanding our knowledge of the historical levels of genetic diversity for *P. falciparum* in a place and period for which very limited information exists.

## Results and discussion

### Presence and abundance of *Plasmodium* spp.

**Figure 1:**
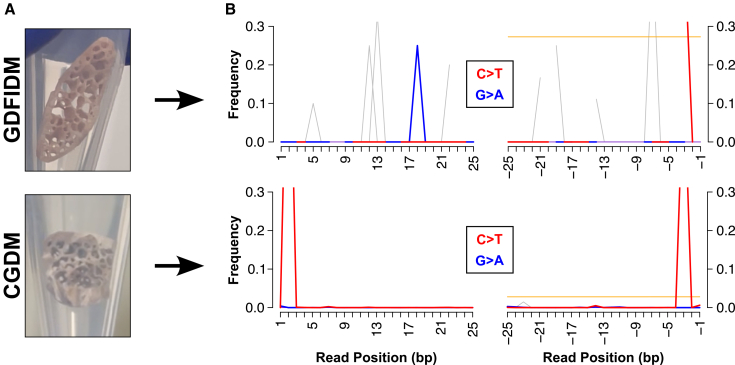
*P. falciparum* mtDNA misincorporation plots derived from target enrichments conducted on the osteological remains of GDFIDM and CGDM (A) Rib fragments of ∼100 mg obtained from GDFIDM and CGDM. (B) DNA misincorporation plots for GDFIDM and CGDM indicating C>T (red) and G>A (blue) transitions at the ends of the reads after mapping the data exclusively against the *P. falciparum* mtDNA reference genome (GenBank: LR605957.1, 3D7 isolate); gray lines reflect all other substitutions, and orange lines represent soft-clipped bases. These plots were generated prior to the filtering of reads based on mapping qualities and mismatch rates (see STAR Methods: “quantification and statistical analysis”).

We extracted DNA from four rib samples, three belonging to GDFIDM and one to CGDM (∼100 mg each; Figure 1A). DNA extracts were enriched for six *Plasmodium* species (*P. falciparum*, *P. vivax*, *P. malariae*, *P. ovale* spp., *P. knowlesi*, and *P. cynomolgi*) through the hybridization of 80-mer probes, designed by Marciniak et al.,^3^ to the mtDNA genomes of these species. DNA libraries were subsequently sequenced on AVITI PE150 runs. Homology searches based on exact *k*-mer alignments^31^ and posterior Bayesian taxonomic reassignments^32^ indicated that out of 2.145 million read pairs sequenced from CGDM, 157,754 (7.4%) were assigned to *Plasmodium* spp. (Table S1); from GDFIDM, out of a total of 10.855 million read pairs across libraries, only 51 (0.0005%) were estimated to be of *Plasmodium* spp. origin (Table S1). These results demonstrate the presence of *Plasmodium* spp. in the osteological remains of both CGDM and GDFIDM, although in much smaller amounts in GDFIDM. The remainder of the classified reads were assigned to contaminant taxa. This is likely due to the presence of human and microbial DNA in the extracts and their successive carry over to the enriched libraries for *Plasmodium* spp.

Competitive mappings of the data against the mtDNA genomes of *P. falciparum* (GenBank: LR605957.1, 3D7 isolate; 5,967 base pairs [bp]) and the other *Plasmodium* species (*P. vivax* [GenBank: NC_007243.1; 5,990 bp], *P. malariae* [GenBank: AB354570.1; 5,968 bp], *P. ovale* spp. [GenBank: AB354571.1; 5,974 bp], *P. knowlesi* [GenBank: NC_007232.1; 5,957 bp], and *P. cynomolgi* [GenBank: AB434919.1; 5,983 bp]) revealed the presence of *P. falciparum* in both CGDM and GDFIDM (Table S2). Specifically, using this mapping method, we recovered 21 fragments totaling 1,110 bp from CGDM and one fragment of 67 bp from GDFIDM that aligned to *P. falciparum* (Table S2). CGDM presented no reads mapped against the other *Plasmodium* species, while from GDFIDM, we additionally recovered one fragment of 43 bp that aligned to *P. malariae* (Table S2).

### Non-competitive mapping against *P. falciparum*

We then mapped the data exclusively against the mtDNA genome of *P. falciparum* (GenBank: LR605957.1, 3D7 isolate) in a non-competitive fashion, recovering 1,865/5,967 bp from CGDM (31.3% mtDNA genome coverage; 3,233× sequencing depth) and 185/5,967 bp from GDFIDM (3.1% mtDNA genome coverage; 0.12× sequencing depth) (Tables S3 and S4). In both cases, the average length of the mapped reads was <70 bp (Table S3), suggesting high amounts of degraded DNA in the targeted sequences and supporting their historical provenance.^33^

Fragment misincorporation plots derived from this process (Figure 1B) illustrate C>T and G>A transitions at the ends of the reads, possibly resulting from postmortem mutations. In this regard, C>T postmortem mutations arise from the deamination of cytosines, each of which is converted into uracil and then incorrectly sequenced as thymines.^34^ Conversely, postmortem cytosine deaminations tend to result in spurious G>A mutations on the complementary strand of DNA during library preparation for sequencing.^35^ Such postmortem mutations are likely to occur at the termini of the reads because this is where the DNA molecule is most exposed to the environment.^36^

All *P. falciparum* sites recovered from GDFIDM were also present in CGDM, with no nucleotide differences between both sequences. However, we excluded the GDFIDM sequence from further analyses given its low coverage and depth. When comparing the CGDM sequence against the reference mtDNA genome of *P. falciparum* (GenBank: LR605957.1, 3D7 isolate), we found two polymorphisms at positions 1,917 (G>T) and 2,708 (C>T). The first polymorphism was found in an intergenic region of the *P. falciparum* mtDNA genome, while the second polymorphism translates to a p.(Thr255Ile) amino acid substitution in the mtDNA cytochrome *c* oxidase subunit 1 gene of this species. We, nonetheless. warrant caution in interpretations concerning the C>T transition leading to the aforementioned amino acid substitution (position 2,708) because this mutation was found near the 3′ end of stacked collapsed reads, and it is likely the result of a postmortem mutation or a base miscall (see Figure S1 for nucleotide substitutions in both GDFIDM and CGDM samples).

**Figure 2:**
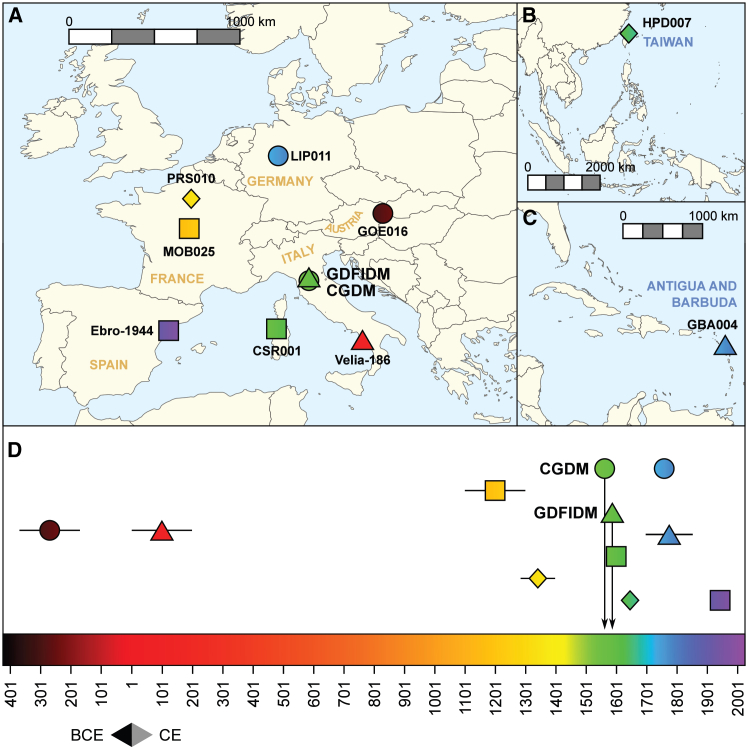
Time and location of ancient *P. falciparum* genetic samples analyzed in this study (A–C) Geographic distribution of ancient *P. falciparum* mtDNA genomes from (A) Europe, (B) Asia, and (C) the Americas, in addition to the *P. falciparum* mtDNA data generated from GDFIDM and CGDM. (D) Estimated ages of the ancient *P. falciparum* samples, with horizontal bars indicating dating ranges. Lines with arrows indicate the years of death for CGDM (1562) and GDFIDM (1587). Data from the samples GOE016, MOB025, PRS010, CSR001, HPD007, LIP011, and GBA004 were generated by Michel et al.^2^ Data from the samples Velia-186 and Ebro-1944 were generated by Llanos-Lizcano et al.^4^ and Gelabert et al.,^23^ respectively. See Table S3 for more details about the ancient samples, including GDFIDM and CGDM.

To determine the geographical origin of the CGDM strain by means of phylogenetic analyses, we assembled a dataset that included published ancient and modern (i.e., last 40 years) *P. falciparum* samples (see Table S3 for sample details). We selected nine samples from three continents (Europe = 7; Asia = 1; Americas = 1; Figure 2), dating from the Iron Age to the 1940s, out of the 15 foregoing publicly available ancient mtDNA sequences,^2^^,^^4^^,^^23^ choosing only samples with high coverage (<10% missingness) across the 1,865 sites defining the CGDM haplotype and excluding some samples because they also included other *Plasmodium* species (see Table S3 and Michel et al.^2^). The modern samples included 31 worldwide sequences from GenBank (Asia = 10; Americas = 7; Africa = 14) and represented unique haplotypes from each geographic location across the same 1,865 sites defining the CGDM haplotype (Table S3). Ultimately, with the addition of multiple *Plasmodium* spp. sequences (Table S3), we used an alignment matrix consisting of 1,694 sites for the analyses after the retention of monomorphic and biallelic sites containing no missing data (i.e., Ns and indels) and no individual heteroplasmies, the latter of which have been documented in different *Plasmodium* species^37^^,^^38^^,^^39^ (see Table S4 for coverages across mtDNA genes; Table S5 for heteroplasmies in the ancient sample set).

**Figure 3:**
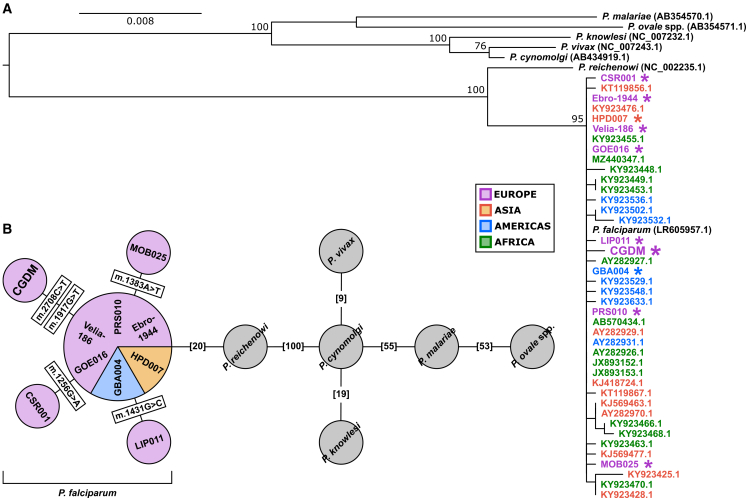
Phylogenetic analyses of the *P. falciparum* mtDNA haplotype from CGDM in relation to ancient and modern *P. falciparum* haplotypes and other *Plasmodium* species (A) Consensus maximum-likelihood phylogenetic tree including CGDM, nine other ancient *P. falciparum* sequences, 31 modern *P. falciparum* sequences, the *P. falciparum* reference, and six different outgroup *Plasmodium* species. Ancient *P. falciparum* sequences are indicated with an asterisk (∗); modern sequences are represented with GenBank accession numbers. Only nodes with bootstrap support values ≥ 70 are shown. The scale bar at the top left reflects substitutions per site. (B) Minimum spanning network from CGDM, nine other ancient *P. falciparum* sequences, and six different outgroup *Plasmodium* species. Hatch marks (rectangles) and numbers inside brackets represent the number of mutational steps between haplotypes (circles). Nucleotide mutations by specific position are shown inside the hatch marks for the *P. falciparum* sequences. Both (A) and (B) were built from an alignment matrix encompassing 1,694 sites; in both cases, colors represent the affiliation to a particular continent as indicated by the caption. Summary statistics of the ancient and modern samples, including geographical location, are presented in Table S3.

A maximum-likelihood phylogenetic reconstruction performed with the *P. falciparum* samples and multiple *Plasmodium* species (Figure 3A) shows that the sequence we retrieved from CGDM positively belongs to the *P. falciparum* clade, with a 95% bootstrap support value. However, this analysis did not allow us to clarify the fine-scale geographical relationships within this clade, as also seen in a previous study.^3^

To gain additional insights into the phylogenetic relationships of the CGDM sequence, we generated a minimum-spanning haplotype network where we focused on the ancient *P. falciparum* samples (Figure 3B). This analysis indicated that the CGDM haplotype is two mutational steps away from a core of samples from Lower Austria (GOE016: 371–176 BCE), Southern Italy (Velia-186: 1^st^–2^nd^ century CE), France (PRS010: 1280–1395), Taiwan (HPD007: 1626–1668), Antigua and Barbuda (GBA004: 1700–1850), and Spain (Ebro-1944: 1940s). This core of ancient samples connected the remainder of the ancient haplotypes, three in total and all from Europe, by one mutational step each, displaying a star-like configuration consistent with a demographic expansion of *P. falciparum* in Europe. Such demographic trends are validated by significantly negative Fu’s *F*_*S*_ (−9.46; *p* < 0.001) and Tajima’s *D* (−1.60; *p* = 0.040) values, reflecting an overall preponderance of singletons and rare haplotypes in the ancient European samples. Additional analyses contemplating the removal of PCR duplicates from the ancient sample set produced similar phylogenetic and demographic results (Figure S2; Tables S4 and S6).

These results suggest that the CGDM strain may have derived from a *P. falciparum* strain already circulating in the Italian peninsula (Velia-186: 1^st^–2^nd^ century CE) and elsewhere in Europe (GOE016: 371–176 BCE; PRS010: 1280–1395). However, other possibilities encompassing a non-European origin for the CGDM strain shall remain open given the scant ancient strain representation from other continents and the limited amount of inter-strain variability found in the short *P. falciparum* sequences we interrogated.

### Conclusions

Overall, our results indicate that CGDM contracted a previously uncharacterized *P. falciparum* strain; this strain might have caused his death in 1562 according to historical accounts consistent with malaria symptoms,^25^ but the presence of the strain itself does not necessarily imply the cause of death. Results from GDFIDM are more ambiguous given the presence of small molecular traces of *P. falciparum* and *P. malariae* in his osteological remains. These traces could either be a false biological signal due to non-species-specific hybridizations in the capture array and minute amounts of DNA template in solution or be a real biological signal suggesting that both species were co-occurring in GDFIDM. We cannot distinguish between these two possibilities. However, it is worth noting that the co-occurrence of different *Plasmodium* species during that time was not uncommon in Europe. For instance, Michel et al.^2^ reported *P. falciparum*-*P. malariae* and *P. falciparum*-*P. vivax* co-occurrences in osteological remains in Belgium from the 15^th^ to 17^th^ century.

Malaria was eradicated in Europe in the early 1970s, but this disease is still present in sub-Saharan Africa, Oceania, South and Southeast Asia, and South America, being responsible for 280 million cases worldwide in 2024 alone,^40^ with *P. falciparum* being the major causal agent. The finding of a previously uncharacterized strain of this species from the Italian Renaissance advances our knowledge of the diversity of strains circulating in a period for which information on this species is very limited, thus expanding our knowledge of *P. falciparum* variation in historical times.

### Limitations of the study

The main limitation of the study is the fact that we could retrieve only relatively small amounts of both short and sparsely distributed mtDNA fragments from *P. falciparum*, even after using an enrichment method to specifically target the mtDNA from this and other *Plasmodium* species in the samples. This was likely due to a series of common factors when dealing with osteoarcheological samples, such as contamination from other organisms, natural degradation, and postmortem mutations, whose impact and extent are difficult to predict *a priori*, often being sample dependent and not necessarily linked to the age of the sample. The short lengths of the retrieved sequences did not allow us to carry out several evolutionary and demographic analyses that could have provided additional insights into the phylogenetic relationship and geographic origin of the Medici *P. falciparum* sample(s). However, these short mtDNA fragments had sufficient historical information to allow us to confirm that (1) the two Medici family members examined in this study were infected with *Plasmodium* parasites; (2) at least for one of them, CGDM, the parasite was clearly *P. falciparum*; (3) the strain of this species was a previously uncharacterized one; and (4) this strain was likely closely related to an ancient European strain. Further sampling of osteological remains of GDFIDM and CGDM will be needed to recover a larger fraction of the previously uncharacterized *P. falciparum* mtDNA genome that we identified in this study.

## Resource availability

### Lead contact

Requests for further information and resources should be directed to and will be fulfilled by the lead contact, Serena Tucci (serena.tucci@yale.edu).

### Materials availability

This study did not generate new unique reagents.

### Data and code availability

• All BAM files derived from the competitive mappings to *Plasmodium* spp. and the non-competitive mappings to *P. falciparum* have been deposited in NCBI BioProject: PRJNA1370089.
• The bioinformatics code used for this study is available in GitHub at https://github.com/AlexanderOchoaH/Analysis-of-Ancient-DNA-Samples.
• The multiple sequence alignment files generated in this study are available in Figshare at https://doi.org/10.6084/m9.figshare.32048529.
• Additional details needed to reanalyze the data can be obtained from the lead contact upon request.

## Acknowledgments

This study was partly funded by the A. Whitney Griswold Faculty Research Fund from 10.13039/100005326Yale University. The study of the Medici remains was authorized by the Superintendent of the Florentine Museums, Professor Antonio Paolucci, as part of the Medici Project. We thank Dr. Monica Bietti, former director of the Medici Chapels Museum, for facilitating the exhumation and field study, Mallory Cox and Dr. Roderick McIntosh for collecting the osteological samples used in this study, the Yale Analytical and Stable Isotope Center for kindly providing liquid nitrogen for the DNA extractions, and Audrey Tjahjadi for helping with the graphical abstract.

## Author contributions

S.T., V.G., and A.C. conceived the study; V.G., G.F., A.F., and G.R. provided samples and contributed to the interpretations of the results; A.O. and S.L.M. performed DNA extractions; A.O. analyzed sequencing data and performed phylogenetic and demographic analyses. P.F.R. developed code for abundance quantifications of *Plasmodium* spp.; S.T. and A.C. supervised the analyses; A.O., S.T., and A.C. drafted the manuscript. All authors have contributed to this work, discussed the results, and critically reviewed and revised the manuscript.

## Declaration of interests

The authors declare no competing interests.

## STAR★Methods

### Key resources table

**Table:** 

REAGENT or RESOURCE	SOURCE	IDENTIFIER
**Biological samples**		

Grand Duke Francesco I de' Medici (GDFIDM; rib sample)	Cappelle Medicee, Florence, Italy	MED11
Cardinal Giovanni de' Medici (CGDM; rib sample)	Cappelle Medicee, Florence, Italy	MED3

**Critical reagents and materials**		

Dabney Deluxe lysis buffer	Dabney and Meyer^41^	Prepared in-house
Proteinase K solution (20 mg/mL)	MP Biomedicals	SKU: 02183988-CF
MinElute PCR purification kit (Buffer PB, Buffer PE, and spin columns)	Qiagen	Catalog No. 28004
DNA extraction protocol	Adapted from Jensen et al.^42^	https://doi.org/10.1038/s41437-022-00510-8
Bait set for the enrichment of *Plasmodium* spp. mtDNA	Designed by Marciniak et al.^3^	D10115MalM2 (available at Daicel Arbor Biosciences)
myBaits High Sensitivity protocol (v5.03)	Daicel Arbor Biosciences	https://arborbiosci.com

**Deposited data**		

BAM files derived from competitive mappings to *Plasmodium* spp. and non-competitive mappings to *P. falciparum*	This study	NCBI BioProject: PRJNA1370089
Bioinformatics code	This study	https://github.com/AlexanderOchoaH/Analysis-of-Ancient-DNA-Samples
Multiple sequence alignments	This study	https://doi.org/10.6084/m9.figshare.32048529

**Software and algorithms**		

Kraken (v2.1.3)	Wood et al.^31^	https://github.com/DerrickWood/kraken2/releases
Bracken (v2.9)	Lu et al.^32^	https://github.com/jenniferlu717/Bracken/releases
PALEOMIX (v1.3.8)	Schubert et al.^43^	https://github.com/MikkelSchubert/paleomix/releases
BLASTN (v2.17.0+)	Altschul et al.,^44^ Camacho et al.^45^	https://www.ncbi.nlm.nih.gov
AdapterRemoval (v2.3.2)	Schubert et al.^46^	https://github.com/MikkelSchubert/adapterremoval
BWA (v0.7.17)	Li^47^	https://github.com/lh3/bwa
mapDamage (v2.2.1)	Jónsson et al.^48^	https://ginolhac.github.io/mapDamage
SAMtools (v1.21)	Li et al.^49^	https://github.com/samtools/samtools/releases
ANGSD (v0.941)	Korneliussen et al.^50^	https://github.com/ANGSD/angsd
PopART (v1.7)	Leigh and Bryant^51^	http://popart.maths.otago.ac.nz
MUSCLE (v3.8)	Edgar^52^	https://drive5.com/muscle/downloads_v3.htm
Geneious (v11.1.5)	Kearse et al.^53^	https://www.geneious.com
BCFtools (v1.21)	Danecek et al.^54^	https://github.com/samtools/bcftools/releases
VCFtools (v0.1.16)	Danecek et al.^55^	https://github.com/vcftools/vcftools
IQ-TREE (v1.6.12	Nguyen et al.^56^	https://iqtree.github.io/release
FigTree (v1.4.4)	Rambaut^57^	http://tree.bio.ed.ac.uk
Arlequin (v3.5.2.2)	Excoffier and Lischer^58^	https://cmpg.unibe.ch/software/arlequin35
Picard (v2.25)	Broad Institute^59^	http://broadinstitute.github.io/picard
Integrative Genomics Viewer (v2.19.2)	Robinson et al.^S1^	https://igv.org

### Experimental model and study participant details

Rib bones from GDFIDM and CGDM (one from each) were collected at the Laboratory of Paleopathology of the University of Pisa (Italy). Specifically, the osteological remains from GDFIDM and CGDM are known as “MED11” and “MED3”, respectively.^60^^,^^61^ At the moment of exhumation from the Medici Chapels (Cappelle Medicee) in Florence, Italy, the metal boxes containing the remains of GDFIDM and CGDM were intact, and the bones were in an excellent state of preservation, but the interior of CGDM’s coffin showed signs of condensation. It should also be considered that these were not the original burials, as the remains were examined and manipulated during previous anthropological studies, and therefore it is not possible to ascertain other possible displacements or damages for DNA preservation.

### Method details

In a dedicated ancient DNA facility at Yale University, we removed surface contamination from the two Medici rib samples using an abrasive rotatory tool. We excised three and one bone fragments of ∼100 mg each from the GDFIDM and CGDM samples, respectively (Figure 1A), and used a cryogenic freezer/mill to grind the bone fragments into fine powder. Following an overnight incubation period where we stored the bone powder in lysis buffer (Dabney Deluxe Buffer^41^ and Proteinase K) at 56°C, we mixed the solution with 9x volumes of Qiagen Buffer PB and used MinElute spin columns and centrifugation to concentrate the DNA extracts. We then alternated between the addition of Qiagen Buffer PE and centrifugation steps to purify the DNA, which was ultimately stored in ∼50 μL of ultrapure DNase/RNase-free water. This protocol for the extraction of DNA from ancient bone samples was adapted from Jensen et al.^42^

DNA extractions were enriched for the mtDNA of six *Plasmodium* species and sequenced at Daicel Arbor Biosciences (Ann Arbor, Michigan). In this regard, 80-mer probes tiled per 20 bp (D10115MalM2 bait set; 3,267 probes in total), designed by Marciniak et al.^3^ from *P. falciparum* (GenBank: NC_002375.1, updated to GenBank: LR605957.1; 5,967 bp), *P. vivax* (GenBank: NC_007243.1; 5,990 bp), *P. malariae* (GenBank: AB354570.1; 5,968 bp), *P. ovale* spp. (GenBank: AB354571.1; 5,974 bp), *P. knowlesi* (GenBank: NC_007232.1; 5,957 bp), and *P. cynomolgi* (GenBank: AB434919.1; 5,983 bp), were used for the enrichments. Library preparation and capture methods were performed by Daicel Arbor Biosciences and were similar to those described in Marciniak et al.,^3^ with a few modifications: library preparations were conducted using a single-stranded procedure, and the hybridization and wash steps for the capture solution were performed at a higher temperature (60°C instead of 55°C) to increase probe specificity to the mtDNA of *Plasmodium* spp. (details in myBaits High Sensitivity protocol v5.03; https://arborbiosci.com). Libraries were sequenced in PE150 runs on an Element Biosciences AVITI sequencer, producing 2.145–4.355 million read pairs per library (Table S1).

### Quantification and statistical analysis

#### Presence and abundance of *Plasmodium* spp

We assessed the presence and abundance of *Plasmodium* spp. in GDFIDM and CGDM using two methods: (1) a combination of Kraken2 v2.1.3^31^ and Bracken v2.9^32^ and (2) competitive, inter-specific mappings in PALEOMIX v1.3.8^43^ followed by BLASTN v2.17.0+^44^^,^^45^ searches of the mapped sequences. For the first method, we used AdapterRemoval v2.3.2^46^ to collapse the reads after eliminating adapters, terminal Ns, and sequences <25 bp. Subsequently, we used Kraken2 with the pre-compiled PlusPFP database (compiled January 27, 2021; https://benlangmead.github.io/aws-indexes/k2) to assign taxonomic labels to the processed reads based on exact alignments of 31-mers. Pointedly, the PlusPFP database contains all the complete genomes in RefSeq of viruses, prokaryotes, protozoa, fungi, plants, and the human reference as of January 27, 2021. We employed Bracken and its Bayesian framework to estimate *Plasmodium* spp. abundances through redistributions of Kraken’s taxonomic inferences; we assumed uniform read lengths of 150 bp and a threshold of 10 reads per taxon above the genus level for abundance estimations.

For the second method, we employed PALEOMIX, a wrapper software including AdapterRemoval, BWA v0.7.17,^47^ and mapDamage v2.2.1^48^ for the processing and simultaneous mapping of the data against the mtDNA genomes of the six *Plasmodium* species used as references for the design of the probes. Briefly, we trimmed the sequences as above, used the BWA “mem” algorithm for mapping the data, and recalibrated base scores with mapDamage. Next, we used SAMtools v1.21^49^ to remove reads with mapping scores <30 and then those with mismatch rates ≥0.02 to their corresponding reference. In ANGSD v0.941,^50^ we created consensus sequences (see Table S2) from the resulting alignment/map files by outputting the base with the greatest effective depth; this step included the removal of bases with scores <30. Finally, we used BLASTN to perform homology searches of the consensus sequences against the NCBI core_nt database (accessed on November 24, 2025).

#### Non-competitive mapping against *P. falciparum*

Following the previously outlined PALEOMIX, SAMtools, and ANGSD procedures but mapping reads exclusively against the *P. falciparum* reference, we obtained consensus sequences from GDFIDM, CGDM, and 15 publicly available ancient samples spanning different time periods and continents (Table S3). However, we excluded the GDFIDM sequence from subsequent analyses given its low coverage and depth (3.1% of *P. falciparum* mtDNA sites recovered; 0.12x sequencing depth). We also excluded three ancient samples due to low-coverage (<10% missingness across the sites recovered from CGDM) and three ancient samples from the 15^th^–17^th^ centuries from present-day Belgium because the data contained either *P. vivax* or *P. malariae* sequences (see Table S3 and Michel et al.^2^).

We integrated the ancient *P. falciparum* dataset with 31 modern (i.e., last 40 years) *P. falciparum* sequences from different locations worldwide (Table S3). The modern sequences reflect unique haplotypes per country across the sites recovered from CGDM, and were defined as such by PopART v1.7^51^ from an original set of 336 sequences (see Table S4 in Llanos-Lizcano et al.^4^). We used MUSCLE v3.8,^52^ as implemented in Geneious v11.1.5,^53^ to align the sequences for phylogenetic inference. The alignment also included the *P. falciparum*, *P. vivax*, *P. malariae*, *P. ovale* spp., *P. knowlesi*, and *P. cynomolgi* reference sequences along with *P. reichenowi* (GenBank: NC_002235.1; 5,966 bp), a recent common ancestor to *P. falciparum* and known to infect chimpanzees, not humans. We further conducted manual missing data (i.e., Ns, indels) realignments, reduced the alignment to the sites recovered from the CGDM sequence, and removed sites containing missing data, >2 alleles, and heteroplasmies. Specifically, we used a combination of BCFtools v1.21^54^ and VCFtools v0.1.16^55^ to detect heteroplasmies in the ancient samples; we validated “heterozygous” genotypes with --minDP 4, --minGQ 18, and multiple counts of reference and alternate alleles each.

We built a maximum-likelihood tree with the ancient *P. falciparum* dataset, the modern *P. falciparum* sequences, and the other *Plasmodium* species in IQ-TREE v1.6.12^56^ after implementing 1,000 ultrafast bootstrap pseudoreplicates, the GTR+F+G4 model of evolution (as selected by the ModelFinder function following Akaike’s Information Criterion), and no declared outgroups to minimize biases for the phylogenetic reconstruction. We polarized the resulting tree in FigTree v1.4.4^57^ using midpoint rooting.

We used PopART to produce a minimum spanning haplotype network with the ancient *P. falciparum* sequences and outgroup *Plasmodium* species. Additionally, we used Arlequin v3.5.2.2^58^ and 1,000 simulations to test for demographic expansions in the ancient European sequences via Fu’s *F*_*S*_ and Tajima’s *D* statistics. Finally, we repeated the phylogenetic and demographic analyses after the detection and removal of PCR duplicates from the ancient sample set using MarkDuplicates in Picard v2.25^59^ and rmdup_collapsed in PALEOMIX; we used this alternative method to also detect heteroplasmies in the ancient sample set and compare results with those derived from the inclusion of PCR duplicates.

## Supplemental information

Document S1. Figures S1 and S2

Data S1. Chemical catalysis checklists

Table S1. Reads sequenced from each DNA library and Kraken2/Bracken hits to *Plasmodium* spp

Table S2. BLASTN hits to *Plasmodium* spp. from GDFIDM and CGDM

Table S3. Metadata and statistics of the *P. falciparum* mtDNA genomes analyzed in this study

Table S4. *P. falciparum* alignment statistics across mtDNA loci

Table S5. *P. falciparum* mtDNA heteroplasmies in ancient samples after the retention of PCR duplicates

Table S6. *P. falciparum* mtDNA heteroplasmies in ancient samples after the removal of PCR duplicates
