# Supplementary material for: Ancient DNA analyses of remains of the Medici family (16th century) provide insights into the genetic variation of Plasmodium falciparum
Source: iScience. 2026 Jun 17;29(7):116371. doi: 10.1016/j.isci.2026.116371 (PMC13310941; doi:10.1016/j.isci.2026.116371)
Supplement: Document S1. Figures S1 and S2 [file mmc1.pdf]

iScience, Volume *29*

Supplemental information

Ancient DNA analyses of remains of the Medici family (16th century) provide insights into the genetic variation of *Plasmodium falciparum*

Alexander Ochoa, Samantha L. Miller, Patrick F. Reilly, Gino Fornaciari, Antonio Fornaciari, Giulia Riccomi, Valentina Giuffra, Adalgisa Caccone, and Serena Tucci

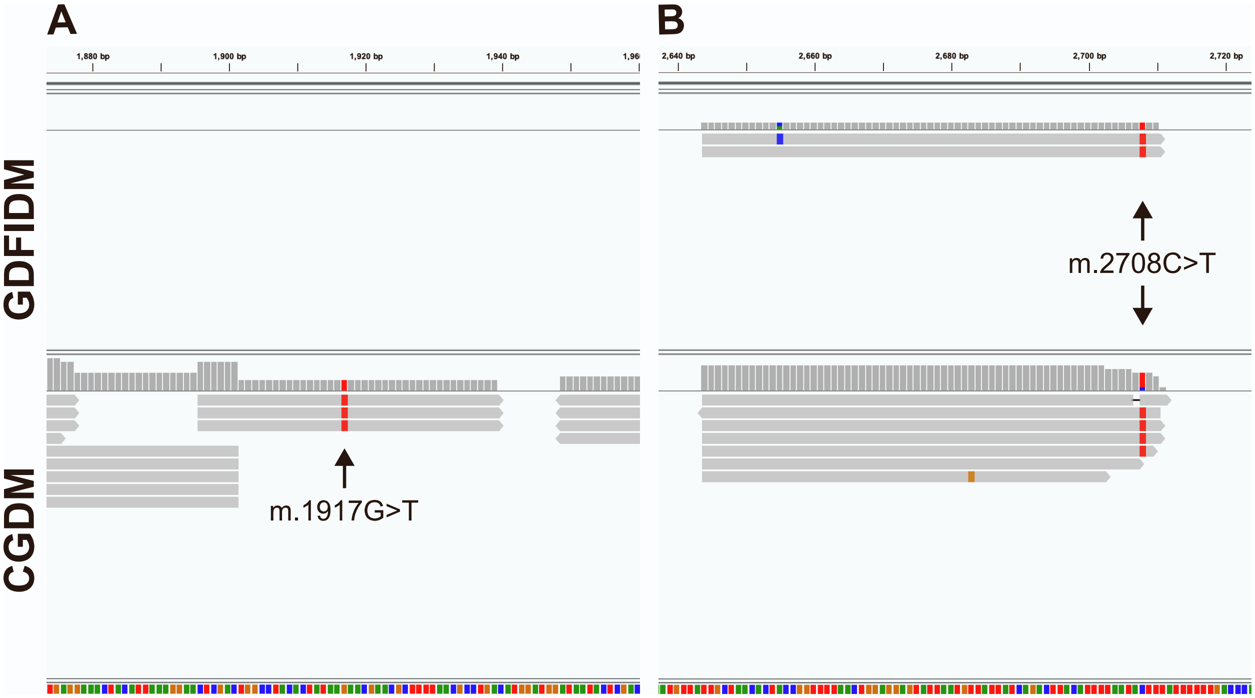

Figure S1. Nucleotide substitutions found in the mtDNA genome of *P. falciparum* from the osteological remains of Grand Duke Francesco I de' Medici (GDFIDM) and Cardinal Giovanni de' Medici (CGDM)

(A) G>T nucleotide substitution in position 1,917 (black arrow) observed in CGDM. This mutation corresponds to a mtDNA intergenic region of *P. falciparum*.

(B) C>T nucleotide substitution in position 2,708 (black arrows) observed in both GDFIDM and CGDM, possibly reflecting a post-mortem mutation or a base miscall. This mutation translates to a p.(Thr255Ile) amino acid substitution in the mtDNA cytochrome c oxidase subunit 1 gene of *P. falciparum*.

Images were generated in Integrative Genomics Viewer v2.19.2 [S1].

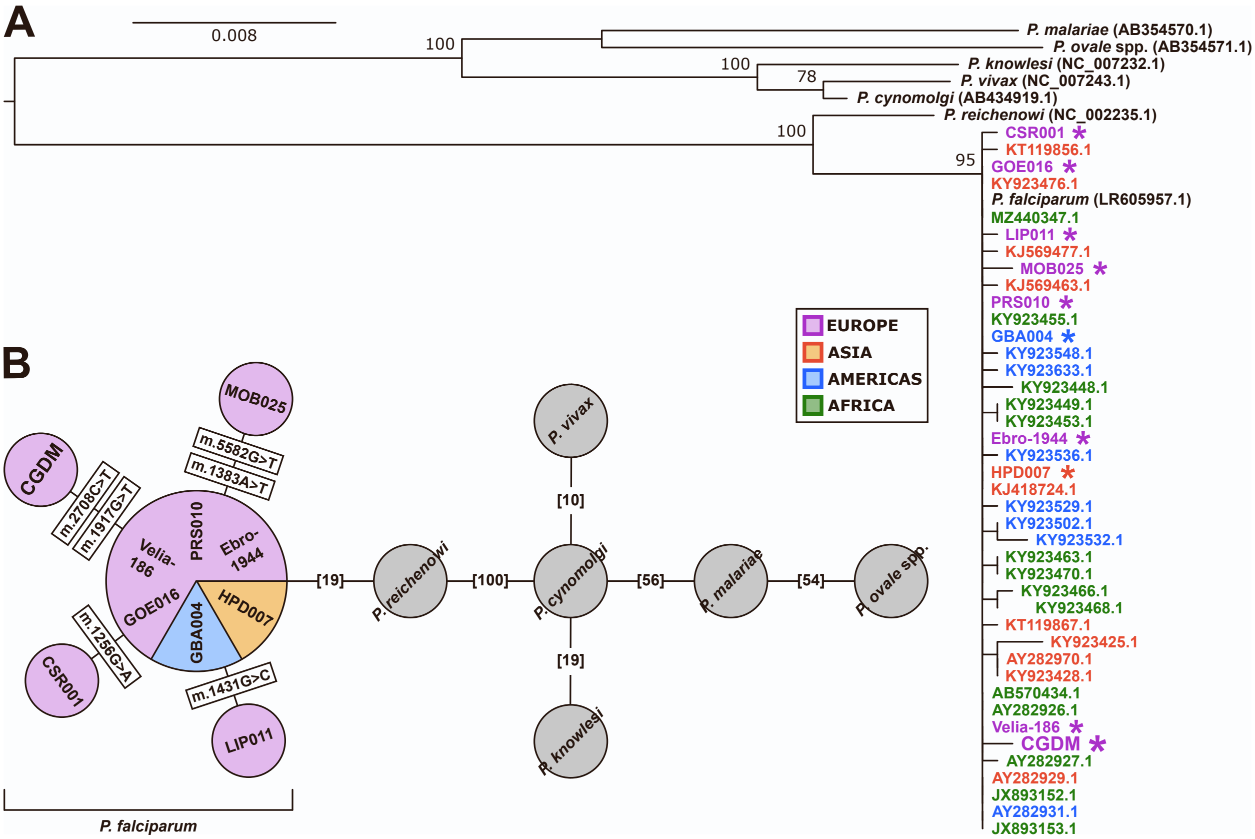

Figure S2. Phylogenetic analyses of the *P. falciparum* mtDNA haplotype from Cardinal Giovanni de' Medici (CGDM) in relation to ancient and modern *P. falciparum* haplotypes and other *Plasmodium* species following the removal of PCR duplicates from the mapped data

(A) Consensus maximum-likelihood phylogenetic tree including CGDM, nine other ancient *P. falciparum* sequences, 31 modern *P. falciparum* sequences, the *P. falciparum* reference, and six different outgroup *Plasmodium* species. Ancient *P. falciparum* sequences are indicated with an asterisk (*); modern sequences are represented with GenBank accession numbers. The GTR+F+G4 model of evolution was used for the phylogenetic reconstruction and only nodes with bootstrap support values ≥70 are shown. The scale bar at the top left reflects substitutions per site.

(B) Minimum spanning network from CGDM, nine other ancient *P. falciparum* sequences, and six different outgroup *Plasmodium* species. Hatch marks (rectangles) and numbers inside brackets represent the number of mutational steps between haplotypes (circles). Nucleotide mutations by specific position are shown inside the hatch marks for the *P. falciparum* sequences.

Both (A) and (B) were built from an alignment matrix encompassing 1,696 sites; in both cases, colors represent affiliation to a particular continent as indicated by the caption. Fu's F_S and Tajima's D values for the ancient European samples were −8.57 ($P < 0.001$) and −1.64 ($P = 0.029$), respectively. Summary statistics of the ancient and modern samples, including geographical location, are presented in Table S3.

SUPPLEMENTAL REFERENCES

S1. Robinson, J.T., Thorvaldsdóttir, H., Winckler, W., Guttman, M., Lander, E.S., Getz, G., and Mesirov, J.P. (2011). Integrative Genomics Viewer. Nat. Biotechnol. *29*, 24–26. (doi:10.1038/nbt.1754)
